# Beyond Triple Therapy: Anti-Type 2 Biologic Therapies for Eosinophilic COPD and Respiratory Comorbidities - a Review of Clinical Evidence

**DOI:** 10.1007/s11882-026-01271-8

**Published:** 2026-04-16

**Authors:** Pier-Valerio Mari, Lorenzo Carriera, Simone Ielo, Loreta Di Michele, Davide Onofrio Fontana, Alberto Ricci, Angelo Coppola, David Selvaggio, Cristiano Caruso, Valeria Gambacorta, Davide Stivalini, Roberto Lipsi, Stefano Baglioni, Raffaele Scala, Eugenio De Corso, Veronica Ojetti

**Affiliations:** 1 Internal Medicine, San Carlo di Nancy Hospital, Rome, 00165 Italy; 2 Faculty of Medicine and Surgery, Università Cattolica del Sacro Cuore, Rome, 00168 Italy; 3 Department of Pulmonology and Sub-Intensive Respiratory Unit, Santa Maria della Misericordia Hospital, Perugia, 06156 Italy; 4 UOC Pneumologia e UTIP, Ospedale San Donato, USL Toscana SudEst, 52100 Arezzo, Italy; 5 Pulmonary Interstitial Diseases Unit, UOSD Interstiziopatie Polmonari Azienda Ospedaliera. S. Camillo-Forlanini, Rome, 00152 Italy; 6 Unit of Geriatrics, Department of Medicine, Università Campus Bio-Medico, Rome, 00128 Italy; 7 Division of Pneumology, Department of Clinical and Molecular Medicine, Sapienza University of Rome, AOU Sant’Andrea, Rome, 00189 Italy; 8 UOC Pneumologia, Ospedale San Filippo Neri-ASL Roma 1, Rome, 00135 Italy; 9 UOS di Malattie dell’Apparato Respiratorio Ospedale Cristo Re, Roma, 00167 Italy; 10 Allergy Unit, Fondazione Policlinico A. Gemelli, IRCCS, Catholic University of the Sacred Heart, Rome, 00168 Italy; 11 Department of Medicine and Surgery, Section of Otorhinolaryngology, Santa Maria della Misericordia Hospital, Perugia, 06156 Italy; 12 UniCamillus International Medical University of Rome, Rome, 00131 Italy

**Keywords:** COPD, Type 2 inflammation, Dupilumab, Mepolizumab, Chronic rhinosinusitis

## Abstract

**Purpose of Review:**

To review current evidence on biologic therapies targeting type 2 inflammation in patients with eosinophilic COPD and respiratory comorbidities who remain uncontrolled despite optimized triple inhaled therapy.

**Recent Findings:**

Recent phase 3 trials have shown that dupilumab provides the most consistent benefit in eosinophilic COPD, reducing exacerbations and improving lung function and quality of life. Mepolizumab has shown a more limited effect, mainly on exacerbation reduction, whereas benralizumab has not demonstrated clear clinical benefit. Blood eosinophil count remains the main biomarker for treatment selection.

**Summary:**

Biologic therapies are advancing precision medicine in COPD, with dupilumab currently showing the strongest overall evidence. Careful patient selection is essential. Broader phenotyping may further improve management, as comorbidities such as chronic rhinosinusitis may contribute to symptom burden and support a multidisciplinary approach. Future studies should clarify long-term outcomes and optimal biologic selection.

## Introduction

Chronic obstructive pulmonary disease (COPD) represents a major global health challenge, ranking as the third leading cause of death worldwide with over 3 million deaths annually and imposing substantial economic burden exceeding $50 billion per year on the United States healthcare system alone [1–3]. The disease is characterized by progressive airflow limitation resulting from airway disease and parenchymal destruction, chronic inflammation, and recurring acute exacerbations that drive disease progression, quality of life deterioration, and mortality [1]. Despite significant advances in bronchodilator therapy with long-acting β2-agonists (LABA) and long-acting muscarinic antagonists (LAMA), and the addition of inhaled corticosteroids (ICS) for selected patients, many individuals continue to experience frequent acute exacerbations (AE-COPD) even while receiving maximal triple inhaled therapy (ICS/LABA/LAMA) [4]. These persistent exacerbations are associated with accelerated lung function decline. Indeed, it has been documented that the reduction in forced expiratory volume in one second (FEV1) persist beyond the acute phase for more than 8 weeks without returning to pre-event baseline values [5]. Moreover, AE-COPD is associated with a markedly increased cardiovascular risk for up to 12 months following the event [6]. The traditional conceptualization of COPD as a disease driven predominantly by neutrophilic inflammation has evolved substantially over the past decade [7]. Emerging evidence demonstrates considerable heterogeneity in underlying inflammatory mechanisms, with approximately 20–40% of COPD patients exhibiting a type 2 (T2) inflammatory endotype characterized by elevated blood and sputum eosinophils, increased levels of T2 cytokines (IL-4, IL-5, IL-13), and enhanced responsiveness to corticosteroid therapy [8]. This T2-predominant phenotype, while representing a minority of the overall COPD population, is clinically significant due to its association with increased exacerbation frequency [9–12]. Increasing recognition of COPD heterogeneity, including T2 inflammatory traits in a subset of patients, has broadened the therapeutic landscape and stimulated evaluation of biologic therapies. While initially established in asthma, these agents have produced encouraging findings in recent COPD trials, albeit with variable responses. Major gaps persist in defining the patients most likely to benefit, given the limited availability of validated biomarkers and the overlap of clinical phenotypes. Future applications may include earlier and potentially disease-modifying treatment aiming at remission [13], combination therapies [14, 15] or even targeted administration during acute exacerbations [16, 17]. This review appraises the current evidence base for biologics in COPD, emphasizing efficacy, safety, ongoing clinical development, and implications for precision and personalized respiratory care.

## Methods

A comprehensive literature search was performed using PubMed, EMBASE and ClinicalTrials.gov, to identify relevant studies published up to January, 2026. Search terms included combinations of keywords such as “COPD”, “eosinophilic COPD,” “biologic therapy,” “monoclonal antibodies,” and names of specific agents. We included only phase 2 or phase 3 randomized controlled trials (RCTs) enrolling adult patients (≥ 40 years) with physician-diagnosed COPD and elevated blood eosinophil counts (e.g., ≥ 150 or ≥ 300 cells/µL, according to trial-specific thresholds), treated with monoclonal antibodies targeting T2 inflammatory pathway. Only studies published in English and available as full-text articles in peer-reviewed journals were considered. We excluded phase 1 trials, observational studies, case reports, case series, small pilot studies, and other non-randomized designs. We also excluded studies focused on asthma or asthma–COPD overlap (ACO/ACOS). We extracted and descriptively summarized available data, then undertook a narrative and critical synthesis; quantitative results were reported as presented in the original studies.

## Pathophysiology of Type 2 Inflammation in COPD

### Overview of Type 2 Inflammatory Cascade

**Fig. 1:**
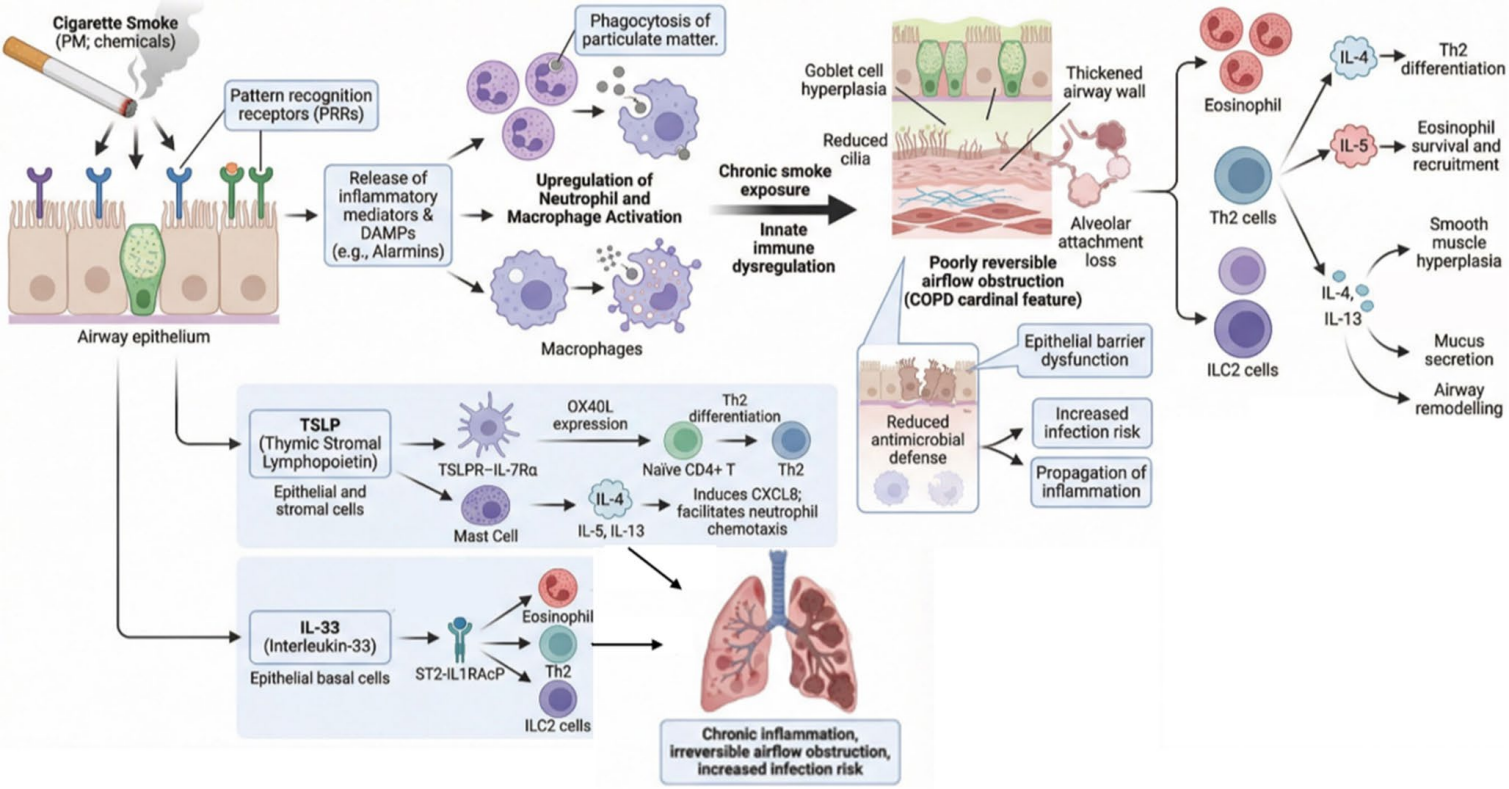
Cigarette smoke activates airway epithelial pattern-recognition receptors, triggering inflammatory mediator release and DAMP/alarmin signaling that recruits neutrophils and macrophages; chronic exposure drives innate immune dysregulation with goblet-cell hyperplasia, impaired ciliary function, airway wall thickening/remodeling, alveolar attachment loss, and emphysema, leading to poorly reversible airflow obstruction and increased infection susceptibility. In parallel, T2 inflammation involves eosinophils, Th2/ILC2 cells, and cytokines IL-4/IL-5/IL-13 (IL-4: Th2 differentiation; IL-5: eosinophil maturation/survival; IL-13: smooth-muscle changes, mucus hypersecretion, remodeling). Epithelial alarmins TSLP and IL-33 amplify T2 and non-T2 responses, promoting downstream cytokine production and eosinophilia; IL-33 can also enhance neutrophilic pathways, induce goblet-cell hyperplasia and impair epithelial repair

T2 inflammation in COPD is initiated by disruption of the airway epithelial barrier caused by cigarette smoke, air pollution, or respiratory infections [18–20]. Epithelial injury triggers the release of alarmins, interleukin-33 (IL-33), interleukin-25 (IL-25), and thymic stromal lymphopoietin (TSLP), which orchestrate both innate and adaptive immune responses [21–23]. Alarmins directly activate type 2 innate lymphoid cells (ILC2), leading to robust production of IL-5 and IL-13 independent of T-cell signaling [24]. Concurrently, dendritic cells capture inhaled antigens and migrate to regional lymph nodes [25]. In lymphoid tissues, dendritic cells present antigens to naïve CD4⁺ T cells. In the presence of IL-4, these cells differentiate into Th2 effector lymphocytes that secrete IL-4, IL-5, and IL-13, thereby amplifying the T2 inflammatory cascade. B cells subsequently undergo class-switch recombination, resulting in IgE production. IL-5 promotes eosinophil differentiation, survival, activation, and recruitment to the airways. IL-4 and IL-13 induce goblet cell metaplasia and mucus hypersecretion, while IL-13 further impairs mucociliary clearance and epithelial barrier integrity [26–28]. Activated eosinophils contribute to tissue damage through the release of cytotoxic granule proteins and the generation of reactive oxygen species [29]. Figure 1 depicts the key steps of the COPD inflammatory cascade, from epithelial activation to downstream effector pathways.

### Blood Eosinophils as a Biomarker

Peripheral blood eosinophil count has emerged as the most accessible and validated biomarker for identifying the T2 inflammatory endotype in COPD patients, with current evidence supporting the use of specific thresholds to guide therapeutic decisions, particularly regarding ICS and eligibility to biologic therapy. The ≥ 150 cells/µL cutoff supports identification of patients likely to benefit from addition of ICS to inhaled therapy [30], while ≥ 300 cells/µL defines a phenotype with even stronger predictive value for ICS response [30] and greater prognostic relevance for future exacerbations, particularly in frequent exacerbators [31–33] (Table 1).

**Table 1:** Predictive and prognostic utility of blood eosinophil thresholds in COPD

Eosinophil threshold	Findings	Effect size	Main clinical implication
≥ 150 cells/µL	Associated with clinically meaningful reduction in exacerbations with ICS [30]	35% reduction in exacerbation risk with ICS (RR 0.65, 95% CI 0.52–0.79) [30]	Predicts clinically relevant ICS benefit
≥ 300 cells/µL	Stronger predictive value for ICS-related exacerbation reduction [30]	39% reduction in exacerbation risk with ICS (RR 0.61, 95% CI 0.44–0.78) [30]	Stronger predictor of ICS response
	Higher exacerbation rates in patients with eosinophils ≥ 300 cells/µL; effect more evident in frequent exacerbators [31, 32, 33]	Adjusted IRR 1.32 (95% CI 1.10–1.63) in COPDGene; 1.22 (95% CI 1.06–1.41) in ECLIPSE [31, 32, 33]	Prognostic value: identifies patients at higher exacerbation risk

Summary of evidence linking blood eosinophil cut-offs (≥ 150 and ≥ 300 cells/µL) to inhaled corticosteroid response and exacerbation risk. Thresholds are shown with key findings and effect sizes from observational cohorts (COPDGene and ECLIPSE) Abbreviations: *ICS* inhaled corticosteroids, *RR* risk ratio, *IRR* incidence rate ratio, *CI* confidence interval

The GOLD 2024–2025 reports integrated blood eosinophil count (BEC) into a treatable-trait approach to guide anti-inflammatory escalation (1). For Group E, LABA+LAMA is the preferred initial regimen; however, triple therapy (LABA+LAMA + ICS) should be considered when BEC ≥ 300 cells/µL, particularly in patients with a high exacerbation burden (e.g., ≥ 2 moderate or ≥ 1 severe exacerbations) [1]. In patients with persistent exacerbations despite optimized inhaled therapy, GOLD also outlines additional step-up options, including biologic therapies in selected patients. For those in the intermediate BEC range (100–300 cells/µL), the expected benefit of ICS is more modest but increases along a continuum, and ICS use is generally favoured when exacerbation risk is prominent. Because BEC shows within-patient biological variability, best practice is to phenotype using repeat measurements obtained during clinical stability rather than relying on a single value. Ultimately, a BEC ≥ 300 cells/µL functions as a high-value, multifaceted biomarker: it identifies patients with a higher likelihood of benefit from ICS-containing regimens [34], supports consideration of targeted biologic strategies in the right clinical context, and marks a population at greater exacerbation risk when inadequately treated. However, consistent with GOLD, BEC should not be used in isolation to predict future exacerbations in an individual patient; its greatest utility is when interpreted alongside symptom burden and exacerbation history within a comprehensive COPD assessment.

### IgE and FeNO in Type 2 COPD

**Fig. 2:**
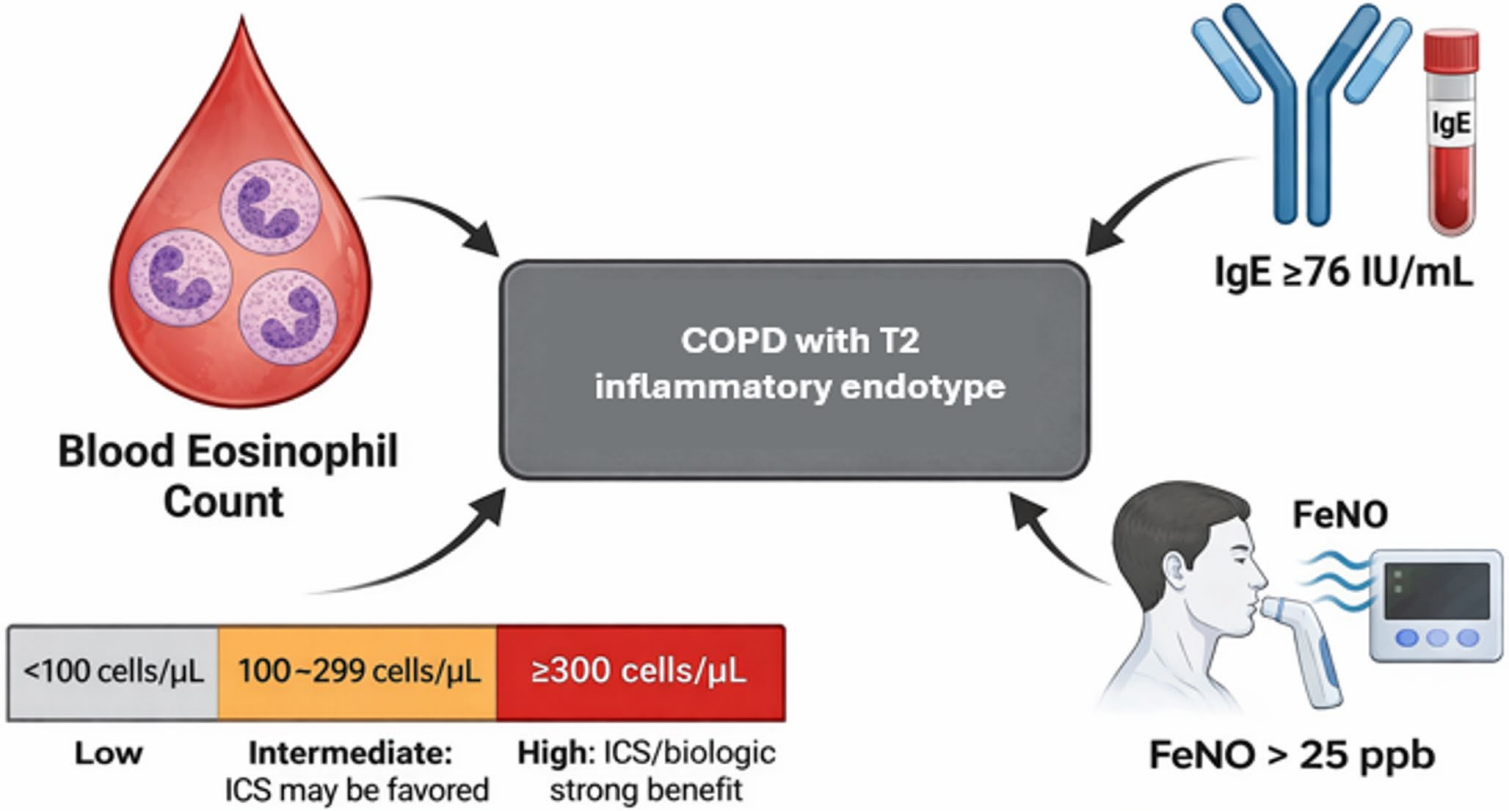
Biomarker profile of T2 inflammation in COPD

While BEC remains the most established and accessible biomarker for identifying T2 inflammation in COPD, emerging evidence supports the potential utility of complementary biomarkers including plasma immunoglobulin E (IgE) and fractional exhaled nitric oxide (FeNO) for enhanced phenotyping and risk stratification. The Copenhagen General Population Study, a large contemporary cohort that included 1,559 individuals with COPD followed over a median of 6.9 years, demonstrated that elevated plasma IgE ≥ 76 IU/mL, the established clinical cutoff for omalizumab treatment in severe asthma, was independently associated with increased risk of severe exacerbation and all-cause mortality in COPD patients, independent of blood eosinophil counts [35]. Interestingly, the study revealed a complex interaction between IgE and eosinophils, where individuals with elevated IgE but normal eosinophil counts (< 300 cells/µL) demonstrated the highest risk for severe exacerbation and mortality, suggesting that IgE may identify a distinct population COPD patients with T2 inflammation. FeNO has demonstrated more limited clinical utility in COPD compared to its well-established role in asthma management. A prospective observational study in a Japanese COPD cohort confirmed that elevated FeNO correlated with clinical features of asthma and eosinophilic airway inflammation, yet baseline FeNO levels were not associated with increased exacerbation rates, hospital admissions, or mortality over 3-year follow-up, contrasting with the robust prognostic performance of blood eosinophil counts [36]. This discordance may reflect fundamental pathophysiological differences between asthma and COPD, including the predominance of small airway disease in COPD where standard FeNO measurement may be less representative of distal inflammation [37]. While blood eosinophil count remains the preferred biomarker for routine clinical practice in COPD due to its accessibility, reproducibility, and validated prognostic value, both plasma IgE and FeNO may serve as complementary tools in selected cases where discordance between clinical phenotype and blood biomarkers raises diagnostic uncertainty, and future research is needed to establish whether integration of multiple biomarkers in composite algorithms might enhance precision in identifying patients most likely to respond to specific anti-inflammatory and biologic therapies, ultimately advancing the paradigm of personalized medicine in eosinophilic COPD management. Figure 2 summarizes the biomarker signature of T2 COPD.

### Mucus Hypersecretion and Airway Plugging

**Fig. 3:**
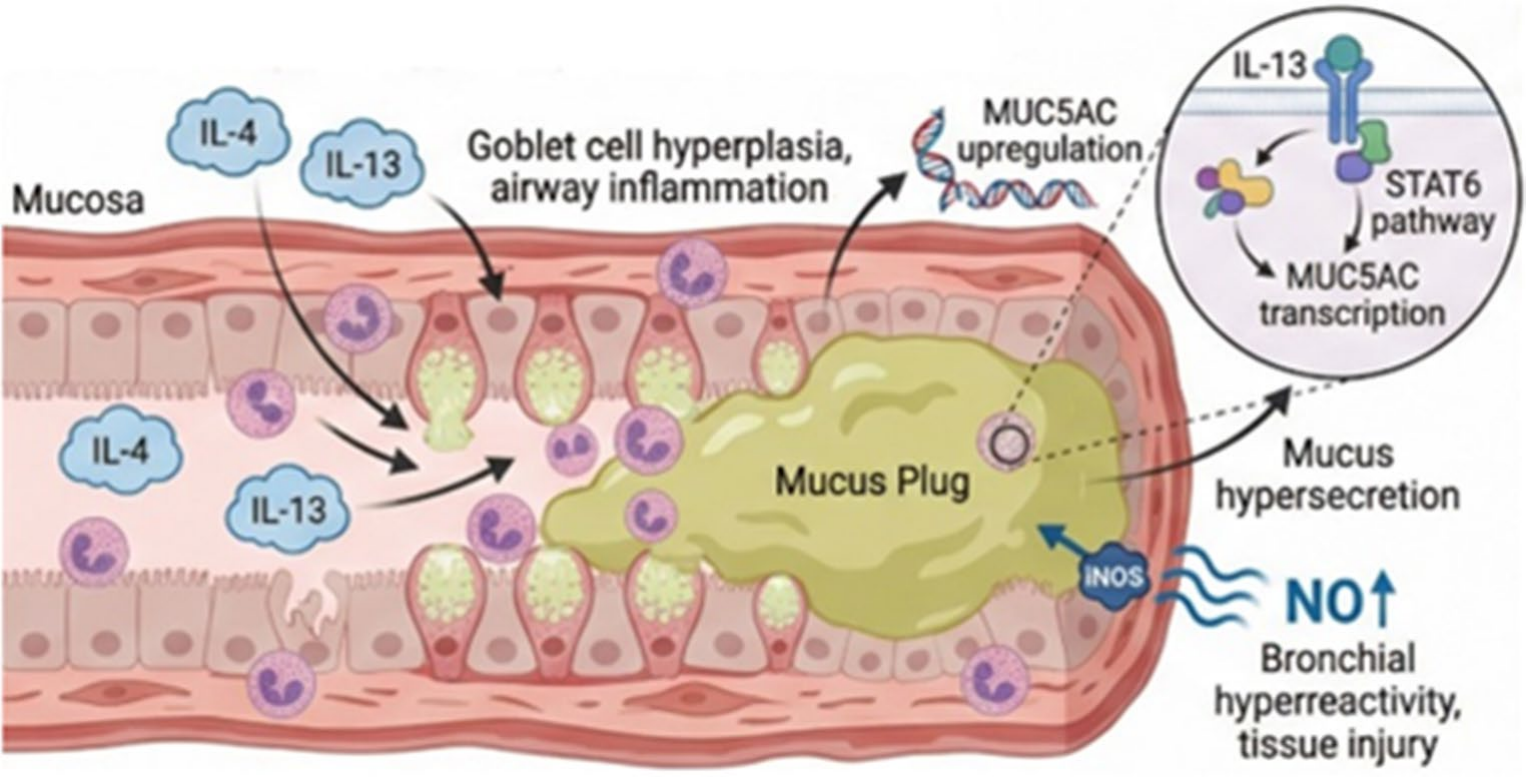
IL-4/IL-13–mediated mucus plugging in eosinophilic COPD. IL-4 and IL-13 promote airway epithelial inflammation and goblet cell hyperplasia, upregulating mucin gene expression, particularly MUC5AC, via downstream signaling (e.g., STAT6), resulting in excessive mucus production and mucus plug formation that contributes to airflow obstruction and increased exacerbation risk

Chronic mucus hypersecretion and airway mucus plugging represent cardinal pathophysiological features of eosinophilic COPD, mediated predominantly by IL-4 and IL-13, which drive goblet cell hyperplasia, mucin gene expression (particularly MUC5AC), and excessive mucus production contributing to airflow obstruction and increased exacerbation risk [38]. IL-13 plays a particularly mechanistic role in promoting mucus hypersecretion and formation of mucus plugs, which is associated with increased mortality in COPD patients [39], while IL-4 and IL-13 together activate inducible nitric oxide synthase leading to airway nitric oxide overproduction, bronchial hyperreactivity, vascular permeability, and tissue damage. Figure 3 illustrates the IL-4/IL-13–driven mechanisms underlying mucus hypersecretion.

Large imaging cohort studies have moved mucus plugs beyond a “chronic bronchitis” symptom correlate by demonstrating their high prevalence in COPD (41–67%) and consistent associations with reduced FEV1, worse symptom burden and quality of life, more frequent exacerbations, hypoxemia and airflow limitation independent of emphysema, and increased all-cause and respiratory mortality, thereby supporting plug burden as a prognostic factor [40]. Importantly, longitudinal CT data further suggest that plugs are dynamic yet often persistent, up to 73% of patients with baseline plugs still exhibit plugs at 5 years, and that both persistent and newly formed plugs are associated with faster FEV1 decline [41]. Its contribution to disease burden and progression has strengthened the rationale for biologics as the most mechanistically precise approach to altering mucus plug formation and persistence. IL-5 blockade targets eosinophil-driven activation, IL-4/IL-13 inhibition limits goblet remodeling and mucus production, and upstream blockade (TSLP, IL-33) may suppress convergent inflammatory inputs. The most convincing proof-of-concept for plug reversibility currently comes from asthma, where dupilumab in VESTIGE reduced CT-assessed plugs and improved functional respiratory imaging measures [42]. It also showed to improve small-airway disease measured with oscillometry [43]. Mepolizumab as well has been associated with reduced plug burden and improved obstruction in severe asthma [44]. In COPD, conventional adjuncts (mucolytics, airway clearance techniques) are widely used to modify mucus properties or facilitate clearance, yet direct evidence that they resolve established plugs is limited. Collectively, the literature supports a perspective in which mucus plugs represent an imaging-defined, prognostically relevant, and potentially modifiable trait.

## PHASE 3 Clinical Trials: Comprehensive Analysis

### DUPILUMAB: Clinical Evidence from the BOREAS and NOTUS Phase 3 Trials

Dupilumab is a fully human monoclonal IgG4 antibody that binds to the interleukin-4 receptor alpha (IL-4Rα) subunit, thereby blocking signaling of both IL-4 and IL-13 [45]. The therapeutic efficacy and safety of dupilumab in eosinophilic COPD were established through two phase 3 multicenter double-blind randomized, placebo-controlled trials, BOREAS and NOTUS [46, 47]. Both trials adopted rigorous inclusion criteria to identify patients with T2 inflammatory COPD most likely to benefit from IL-4/IL-13 pathway blockade: age ≥ 40 years, post-bronchodilator FEV₁/FVC < 0.70 with FEV₁ 30–70% predicted, blood eosinophils ≥ 300 cells/µL at screening, history of ≥ 2 moderate or ≥ 1 severe COPD exacerbation in the previous 12 months (with at least one exacerbation occurring while on triple therapy), chronic productive cough for ≥ 3 months consistent with chronic bronchitis phenotype, and smoking history ≥ 10 pack-years as current or former smoker. Critically, both trials excluded patients with history of asthma, clinical features suggestive of asthma-COPD overlap (ACO), or other chronic lung diseases. Eligible patients were randomized 1:1 to receive either dupilumab 300 mg subcutaneously every 2 weeks plus continuation of triple inhaler therapy or placebo plus triple therapy for the 52-week treatment period. A pooled analysis of these studies, that included all 1,874 randomized patients (938 assigned to dupilumab, 936 to placebo), demonstrated clinically meaningful and statistically significant benefits across multiple outcomes [48]. For the primary endpoint of annualized rate of moderate or severe exacerbations over 52 weeks, dupilumab reduced exacerbations to 0.794 events per year compared to 1.156 events per year with placebo, yielding a rate ratio of 0.687 (95% CI 0.595–0.793, *p* < 0.0001), representing a 31% relative risk reduction and an absolute risk reduction of 0.362 events per year. Notably, the effect of dupilumab on exacerbations was greater (45% reduction) in patients with higher FeNO levels (> 20ppb) [48]. Lung function improvements were rapid and sustained throughout the treatment period, with prebronchodilator FEV₁ increasing at week 12 and week 52 (mean differences 0.083 L and 0.073 L, respectively; both *p* < 0.0001). Dupilumab demonstrated also consistent improvements in health-related quality of life and symptom burden, reducing St. George’s Respiratory Questionnaire (SGRQ) total score by 3.4 points (95% CI -5.0 to -1.8, *p* < 0.0001). Pre-specified subgroup analyses revealed important insights into biomarker-guided patient selection and treatment optimization, demonstrating that patients with higher FeNO experienced greater treatment effects in terms of exacerbation rate ratio and FEV₁ improvement. Treatment benefits remained consistent across current and former smokers, addressing concerns about differential efficacy based on active smoking status, while patients with higher baseline blood eosinophil counts demonstrated greater absolute benefit. The pooled safety analysis demonstrated a favorable safety profile consistent with dupilumab’s established safety experience in other T2 inflammatory diseases.

### MEPOLIZUMAB: Clinical Evidence from the METREX, METRO, MATINEE Phase 3 Trials

Mepolizumab is a humanized monoclonal IgG1 antibody that binds to IL-5 with high affinity, preventing IL-5 from binding to its receptor on eosinophils. This blocks IL-5-mediated signaling, reduces eosinophil production from bone marrow, impairs eosinophil survival, and decreases tissue eosinophil recruitment [49]. The therapeutic potential of mepolizumab in eosinophilic COPD was evaluated through three pivotal phase 3 trials, METREX [50], METREO [50] and MATINEE [51], which collectively enrolled over 2,500 patients. The initial trials, METREX and METREO, employed broader enrollment criteria with blood eosinophil thresholds of ≥ 150 cells/µL at screening or ≥ 300 cells/µL in the previous 12 months. Eligible patients were randomized 1:1 in METREX to receive mepolizumab 100 mg or placebo SC, and 1:1:1 in METREO to receive mepolizumab 100 or 300 mg, or placebo SC every 4 weeks for 52 weeks. METREX demonstrated a statistically significant 18% exacerbation reduction (rate ratio 0.82, 95% CI 0.68–0.98, *p* = 0.04) while METREO showed a non-significant trend (rate ratio 0.86, 95% CI 0.70–1.06, *p* = 0.16), highlighting the importance of patient selection and baseline eosinophil levels in predicting treatment response. The subsequent MATINEE trial implemented more restrictive enrollment criteria aligned with the dupilumab trials (blood eosinophils ≥ 300 cells/µL at screening, ≥ 2 moderate or ≥ 1 severe exacerbations despite triple therapy for ≥ 3 months) and demonstrated a 21% exacerbation reduction (rate ratio 0.79, 95% CI 0.66–0.94, *p* = 0.01) with a number needed to treat of 5 patients, establishing consistent exacerbation benefits in highly eosinophilic COPD patients. However, a critical and consistent limitation emerged across all three trials: mepolizumab failed to demonstrate statistically significant or clinically meaningful improvements in lung function, with prebronchodilator FEV₁ changes ranging from only + 10 to + 20 mL compared to placebo at week 52, far below the minimal clinically important difference of 100 mL and representing a stark contrast to the robust improvements observed with dupilumab in BOREAS and NOTUS. Similarly, patient-reported outcomes including SGRQ showed no statistically significant improvements across the mepolizumab trials, indicating that despite modest reductions in exacerbation frequency, IL-5 pathway blockade did not translate into meaningful improvements in daily symptom burden, functional limitations, or health-related quality of life. The safety profile was consistently favorable across all trials, with overall adverse event rates comparable to placebo and mepolizumab inducing profound eosinophil depletion without increased infection risk.

### BENRALIZUMAB: Clinical Evidence from the GALATHEA and TERRANOVA Phase 3 Trials

Benralizumab, a monoclonal antibody targeting the IL-5 receptor alpha (IL-5Rα) and inducing direct eosinophil depletion through antibody-dependent cell-mediated cytotoxicity (ADCC) [52], was evaluated in two phase 3 trials, GALATHEA (NCT02138916) and TERRANOVA (NCT02155660) [53], which investigated different dosages of the drug administered subcutaneously every 8 weeks in patients with COPD and blood eosinophils ≥ 220 cells/µL, therefore representing a lower eosinophil threshold compared to the mepolizumab and dupilumab trials. Both trials were completed but failed to meet their primary endpoints, demonstrating only a non-significant reduction in annualized exacerbation rates compared to placebo. Secondary endpoints similarly failed to demonstrate clinically meaningful benefits, with small improvements in prebronchodilator FEV₁ and no significant improvements in patient-reported quality of life measures. Several potential explanations for the negative trial results have been proposed, including the possibility that the lower eosinophil threshold of ≥ 220 cells/µL compared to ≥ 300 cells/µL used in successful trials may have diluted the treatment effect by including patients with insufficient T2 inflammatory burden, that complete eosinophil depletion via ADCC mechanism may not provide therapeutic advantages over IL-5 ligand blockade in COPD despite theoretical benefits in other eosinophilic conditions, that the dosing frequency of every 8 weeks may be suboptimal for maintaining adequate pathway suppression in chronic airway disease, and that patient selection criteria may require higher eosinophil thresholds combined with more stringent phenotypic characterization to identify responders. The RESOLUTE study (NCT04053634), currently ongoing, failed as well its primary endpoint i.e. annualized rate of moderate to severe COPD exacerbations [54].

**Fig. 4:**
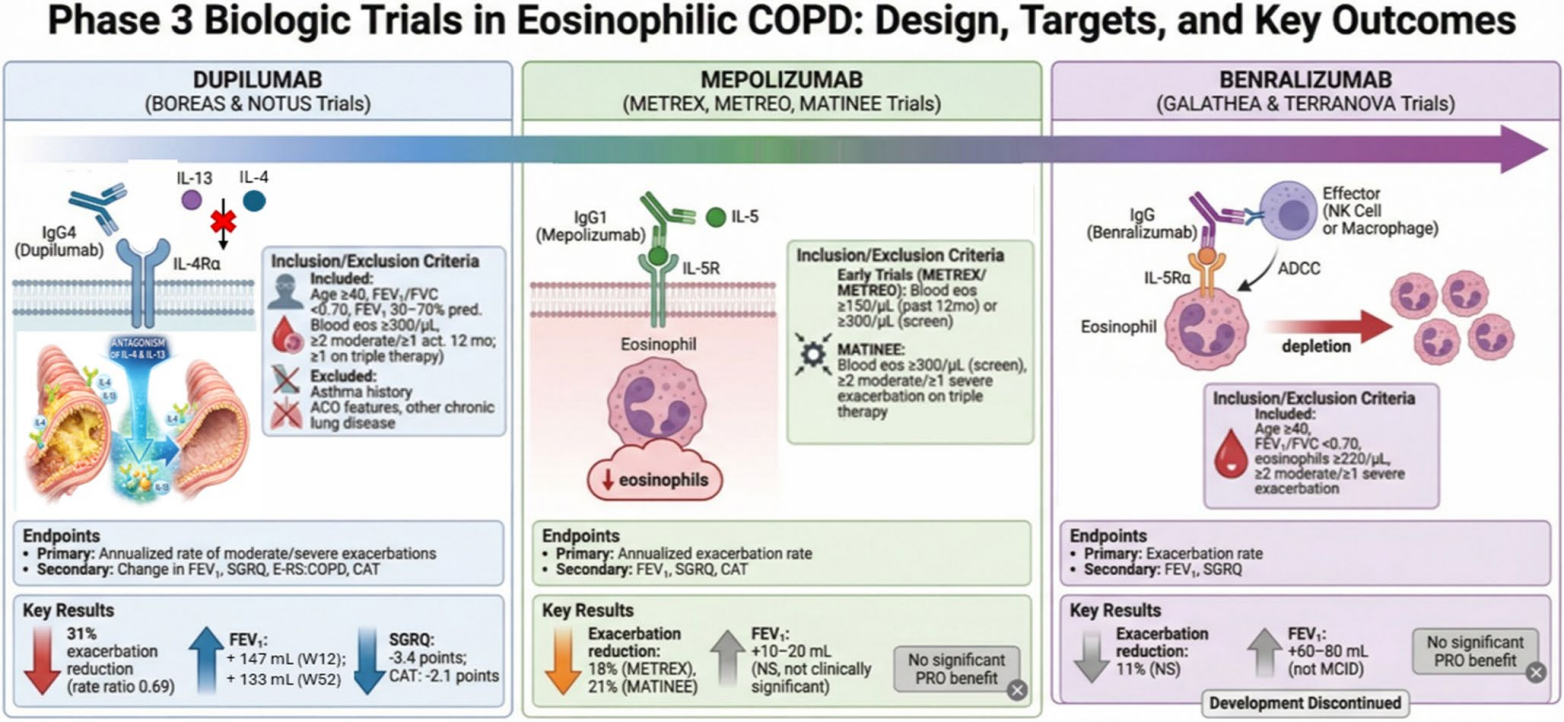
Schematic summary of pivotal phase 3 programs evaluating biologics across eosinophilic COPD populations: dupilumab (anti–IL-4Rα; blocks IL-4/IL-13 signaling; BOREAS/NOTUS), mepolizumab (anti–IL-5; METREX/METREO/MATINEE), and benralizumab (anti–IL-5Rα; eosinophil depletion via ADCC; GALATHEA/TERRANOVA)

Figure 4 provides a comparative overview of phase 3 biologic trials in eosinophilic COPD, highlighting therapeutic targets, eligibility criteria, and key clinical outcomes.

### TEZEPELUMAB: Clinical Evidence from the Course Study

Tezepelumab targets TSLP, an epithelial alarmin that initiates T2 inflammation upstream of IL-4, IL-5, and IL-13 [55]. COURSE [56] was a phase 2a, multicenter, randomized, double-blind, placebo-controlled trial evaluating tezepelumab in patients with moderate-to–very severe COPD (NCT04039113). Overall, 333 participants were randomized to tezepelumab (*n* = 165) or placebo (*n* = 168). Over 52 weeks, the annualized rate of moderate or severe exacerbations was 1.75 with tezepelumab and 2.11 with placebo (rate ratio 0.83 [90% CI 0.64–1.06]; *p* = 0.1). Although the primary endpoint was not met in the overall population, a clear signal of greater benefit emerged with increasing BEC. In a post hoc analysis [57, 58] restricted to patients with BEC ≥ 150 cells/µL, tezepelumab was associated with a statistically significant reduction in moderate or severe exacerbations, apparently consistent across baseline clinical characteristics (FeNO, smoking status, and prior exacerbation history). Mechanism rationale remains strong despite modest phase 2 results, and phase 3 study NCT06883305 [59] is currently recruiting.

## Clinical Implementation: Patient Selection Algorithm Proposal

Patient selection should follow a structured, stepwise pathway that confirms (1) an appropriate clinical COPD population with persistent exacerbation burden despite guideline-based therapy, (2) a biologically plausible type 2–high signal, and (3) patient-informed feasibility and preferences.

- Step 1 - Identify appropriate candidates. Begin with patients with physician-diagnosed COPD confirmed by spirometry (post-bronchodilator FEV₁/FVC < 0.70) and at least moderate airflow limitation (FEV₁ <80% predicted). The target population is “exacerbation-prone” despite optimized care: prioritize individuals with ≥ 2 moderate exacerbations or ≥ 1 severe exacerbation in the previous 12 months while receiving optimized triple inhaled therapy (ICS/LABA/LAMA) for at least 3 months, with documented good adherence and clinically meaningful symptoms (e.g., CAT ≥ 10 or mMRC ≥ 2). Before proceeding, rule out situations in which biologics are not appropriate or evidence is limited: asthma or asthma–COPD overlap, active pulmonary infection, other chronic lung diseases, immunosuppressive conditions, malignancy under active treatment, and pregnancy or breastfeeding.
- Step 2 - Confirm the inflammatory endotype with biomarkers. For eligible candidates, obtain a peripheral blood eosinophil count as the primary, pragmatic biomarker. A threshold of ≥ 300 cells/µL supports a type 2–high inflammation and triggers consideration of biologic therapy. In contrast, if eosinophils are consistently < 300 cells/µL, expected benefit is reduced and biologics are less likely to be effective, prompting optimization of non-biologic strategies instead. Where available, additional biomarkers can strengthen endotype confidence and help refine expectations: FeNO ≥ 20–25 ppb supports type 2 inflammation and may indicate greater likelihood of response, particularly for dupilumab, while elevated total serum IgE provides supportive (though non-specific) evidence of type 2 biology. Sputum eosinophils ≥ 3%, if obtainable, offers direct confirmation of airway eosinophilia.
- Step 3 - Move to shared decision-making and treatment choice. For patients meeting clinical criteria and demonstrating an eosinophilic/type 2 signal, translate eligibility into a practical, patient-centered plan. Discuss the anticipated magnitude of benefit (approximately ~ 30% reduction in exacerbations, with potential improvement in lung function and quality of life), the treatment logistics (subcutaneous administration q2 weeks for dupilumab or q4 weeks for mepolizumab), and the expected duration (often long-term in practice, acknowledging that robust long-term data are mainly limited to ~ 1–2 years). Review safety and tolerability (generally favorable, with common events such as injection-site reactions and nasopharyngitis) and position biologics within the broader therapeutic landscape by explicitly considering alternatives or add-ons (continued optimization of inhaled therapy, roflumilast or azithromycin in selected patients, and pulmonary rehabilitation). The final decision should balance biologic plausibility, exacerbation risk, comorbidities, feasibility, and the patient’s goals and preferences.

**Fig. 5:**
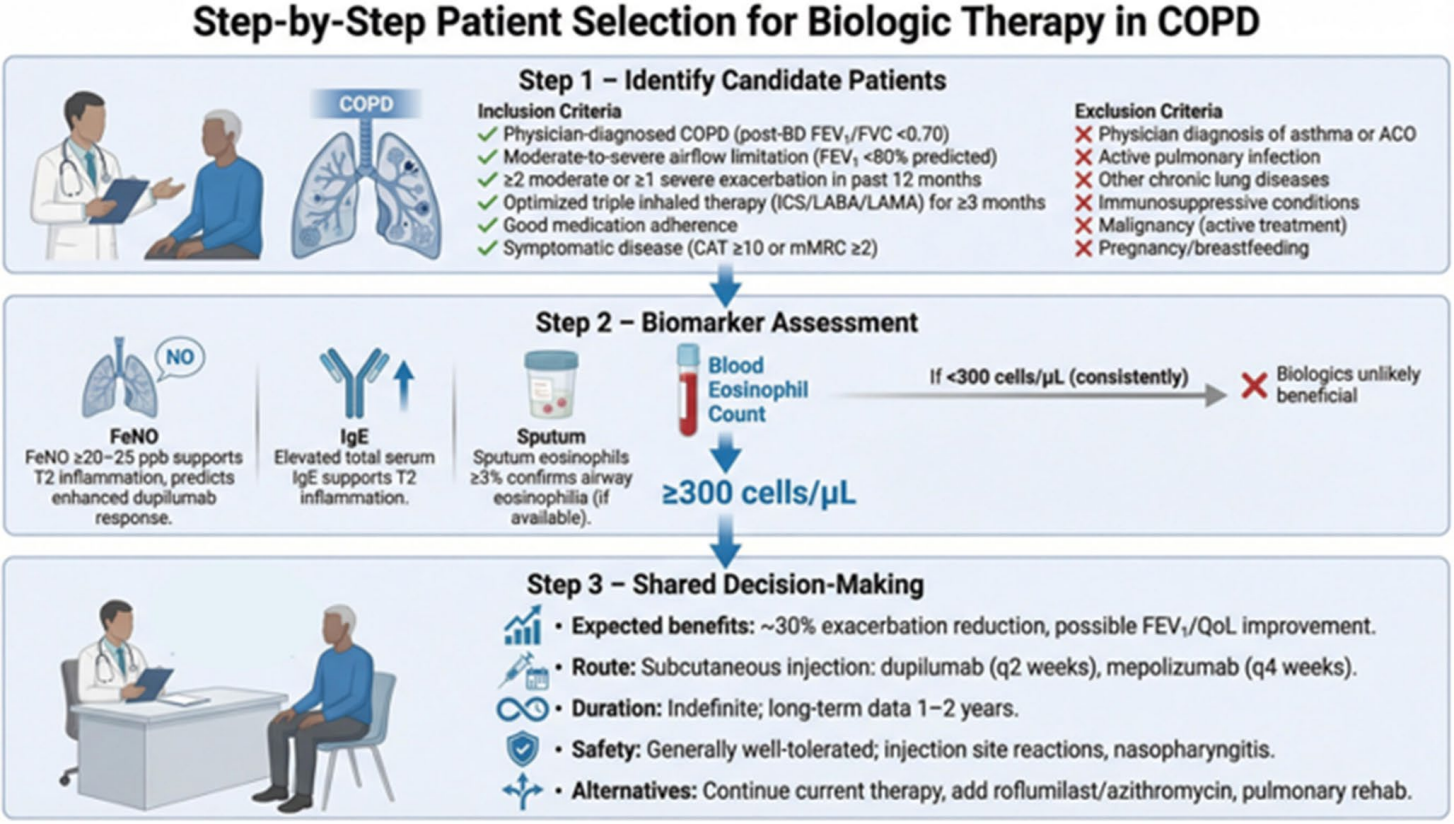
Patient selection algorithm for biologic therapy in COPD

A pragmatic, stepwise algorithm to identify COPD patients most likely to benefit from biologic therapy based on clinical eligibility and biomarker evidence of type 2 inflammation is presented in Fig. 5.

### Future Directions in Biologic Therapy for COPD

Biologics targeting the IL-33 pathway, either by neutralizing IL-33 itself or by blocking its receptor ST2 are emerging as plausible candidates for COPD treatment, given the role of IL-33 as an epithelial “alarmin” released after airway injury and its capacity to amplify downstream inflammation. Within this landscape, the maturity of the evidence differs across agents, with signals that appear subgroup-dependent and, in several cases, discordant across trials, reinforcing the need for careful phenotyping and validated responder biomarkers. Itepekimab, an IL-33-directed mAb, has been associated with efficacy signals primarily in former smokers. It reduced moderate or severe acute exacerbations by 27% compared with placebo after 52 weeks in AERIFY-1. However, itepekimab did not decrease exacerbations after 52 weeks in AERIFY-2, despite showing benefit earlier in the study [60]. Tozorakimab is another IL-33–neutralizing mAB. In phase 2a study (FRONTIER-4; NCT04631016) [61], tozorakimab showed encouraging signals, particularly for lung function, while exacerbation outcomes were more difficult to interpret due to typical phase 2 constraints (including sample size and event rates). Its phase 3 programme includes two pivotal studies, OBERON (NCT05166889) [62] and TITANIA (NCT05158387) [63], both evaluating efficacy and safety in symptomatic COPD with exacerbation history, alongside the PROSPERO extension study (NCT05742802) [64], designed to assess longer-term outcomes in participants completing the parent trials. Astegolimab (anti-ST2) blocks the IL-33 receptor. In the phase 2a COPD-ST2OP (NCT03615040) trial [65], astegolimab did not significantly reduce exacerbation rates, but did demonstrate improvements in patient-reported health status, supporting biological activity without clear exacerbation prevention in the overall cohort. In later development, statistically significant exacerbation reduction was suggested in the phase 2b ALIENTO trial [66], whereas the phase 3 ARNASA (NCT05595642) study reported a numerical reduction that did not reach statistical significance, again underscoring variability across studies and the importance of defining the right target population [67]. Beyond IL-33/ST2, other cytokine-targeted approaches continue to be explored. For example, lebrikizumab (anti-IL-13), already analyzed in asthma, has also been evaluated in COPD (e.g., NCT02546700) [68], reflecting interest in airway remodelling and mucus biology, although its clinical role in COPD remains to be defined.

### Comorbidities and Multidisciplinary Care in COPD: Chronic Rhinosinusitis and the “United Airways” Concept

Comorbidity management has become a core component of COPD care, as patients increasingly present with complex multimorbidity profiles in which symptoms, exacerbation risk, and quality of life are often influenced by conditions extending beyond the lower airways. Medical conditions that frequently coexist with COPD include cardiovascular diseases, lung cancer, osteoporosis, depression/anxiety, and gastroesophageal reflux disease [69]. Increasing attention has also been directed toward the coexistence of upper and lower airway disease, captured by the concept of the “united airways” [70]. Although this paradigm is well established in atopic conditions, pan-airway involvement can also occur in the absence of atopy. In COPD the united airways model proposes that shared exposures, including cigarette smoke, allergens, and other irritants, can induce parallel inflammatory responses in both the nasal and bronchial mucosa [71]. Upper airway involvement in COPD is suggested by the high prevalence of sinonasal complaints. Håkansson et al. have reported that approximately 75–88% of COPD patients experience daily nasal symptoms [70]. Across cohorts, the most commonly reported symptoms include nasal discharge, nasal obstruction, sneezing, and anosmia/hyposmia [71]. Only a limited number of studies have examined the relationship between COPD and sinonasal disease, but available data suggest that Chronic rhinosinusitis (CRS) is not uncommon in COPD and may be clinically under-recognized. In a large Swedish cohort, CRS was reported in 15.3% of ever-smokers with COPD and lower-airway symptoms [72]. In addition, a cross-sectional study of 222 COPD patients found a CRS prevalence of 22.5%, and notably 82% of CRS cases were previously undiagnosed [73]. Comorbid CRS was associated with worse quality of life, and more frequent daily nasal symptoms correlated with poorer overall health status [74]. Moreover upper airway symptoms appear to increase over time in COPD and are associated with the frequent-exacerbator phenotype [71]. This raises the possibility that untreated sinonasal disease may contribute to exacerbation susceptibility. Singh et al. [75] showed in fact that COPD patients with rhinitis had significantly higher 30-day readmission rates compared with COPD patients without rhinitis. Notably, upper airway symptoms have been associated with bronchial and systemic eosinophilia [76]. Collectively, these findings suggest that CRS may be under-recognized during routine COPD assessments and warrants greater attention within a multidisciplinary framework, given the potential relevance of CRS to COPD symptoms, exacerbations, and overall outcomes. Despite these signals, COPD-related sinonasal disease remains incompletely characterized with respect to symptoms, endoscopic findings, and underlying inflammation. Future studies should clarify upper-airway inflammatory endotypes in COPD and determine whether biologic therapy in frequent-exacerbator Type 2 COPD patients can also improve sinonasal symptoms and related outcomes, alongside lower-airway endpoints.

## Discussion

Monoclonal antibodies targeting T2 inflammation represent a paradigm shift toward precision medicine in COPD management, offering mechanism-based, phenotype-specific interventions for patients with eosinophilic disease who remain uncontrolled despite maximal standard treatment. This comprehensive narrative review synthesizes evidence from phase 3 randomized controlled trials investigating monoclonal antibodies in selected patients with T2 inflammatory COPD, highlighting clinical benefits and differences in efficacy profiles between biologic agents, that have distinct targets within the same inflammatory pathway. Dupilumab, through dual IL-4 and IL-13 blockade, demonstrates the most comprehensive clinical benefit profile in eosinophilic COPD with chronic bronchitis, with pooled analysis of BOREAS and NOTUS showing 31% exacerbation reduction (rate ratio 0.687, *p* < 0.0001, NNT = 3), clinically meaningful lung function improvements and significant improvements in patient-reported outcomes. This comprehensive efficacy across inflammation, mucus hypersecretion, airway remodeling, and symptom burden represents the strongest evidence base among evaluated biologics and has earned regulatory approval as the first targeted biologic therapy for COPD. In contrast, IL-5 pathway targeting agents revealed more limited efficacy. Mepolizumab demonstrated 21% exacerbation reduction (rate ratio 0.79, *p* = 0.01, NNT = 5) in MATINEE but failed to improve lung function or quality of life across METREX, METREO and MATINEE trials, suggesting that selective eosinophil depletion alone does not adequately address mucus hypersecretion, airway remodeling, and epithelial pathology driving persistent obstruction and symptoms. Benralizumab trials (GALATHEA, TERRANOVA) failed to demonstrate significant exacerbation reduction despite complete eosinophil depletion. The differential efficacy profiles across biologic agents raise critical mechanistic questions. Clinically meaningful lung function improvements were observed with dupilumab, while mepolizumab failed to demonstrate considerable functional gains despite achieving exacerbation reduction, likely reflecting the pleiotropic effects of IL-4/IL-13 on mucus hypersecretion, goblet cell metaplasia, and mucus plug formation, mechanisms directly impeding airflow. An important methodological consideration concerns the potential influence of background triple inhaled therapy on FEV₁ response magnitude, with near-complete baseline bronchodilation potentially creating a “ceiling effect” limiting additional functional improvement achievable through anti-inflammatory intervention. Blood eosinophil count ≥ 300 cells/µL has emerged as the validated biomarker for identifying patients most likely to benefit from biologic therapy, with this higher threshold demonstrating superior predictive value compared to lower cutoffs (≥ 220 cells/µL used in failed benralizumab trials). Measurement on two separate occasions during clinical stability is recommended to account for temporal variability. Current recommendations suggest measuring eosinophils at least 4 weeks after exacerbations and ideally 6–8 weeks after systemic corticosteroid completion, with patients on stable inhaled corticosteroids acceptable as these produce less pronounced systemic suppression. Complementary biomarkers including plasma IgE (≥ 76 IU/mL) and FeNO (≥ 20 ppb) may provide additional patient selection value, with enhanced dupilumab efficacy observed in patients with elevated FeNO. However, active smoking introduces complex confounding factors, particularly affecting FeNO measurements. Smoking substantially suppresses FeNO through direct nitric oxide scavenging, downregulation of inducible nitric oxide synthase, and altered epithelial function, potentially limiting FeNO utility as a complementary biomarker in current smokers. BOREAS and NOTUS included approximately 30% current smokers and 70% former smokers, with subgroup analyses demonstrating consistent dupilumab efficacy across smoking status, suggesting active smoking does not substantially impair therapeutic response despite biomarker effects. Whether smoking cessation following biologic initiation enhances responses remain incompletely characterized.

Looking ahead, the priorities are clear. Longer-term follow-up beyond 52 weeks is needed to determine whether clinical benefits are sustained, to identify any late-emerging safety signals, and to better define the durability of treatment response. In addition, it remains essential to establish whether biologic therapy provides benefits beyond exacerbation reduction, including potential effects on FEV₁ decline and survival. Equally important is learning how to use these agents over time: are they lifelong therapies, can they be stopped once disease is controlled, or might they be deployed intermittently? Progress will also depend on better patient selection through integrated biomarker strategies combining eosinophils, FeNO and IgE rather than relying on any single marker. Comparative head-to-head studies are needed to clarify biologic drug choice within the T2 inflammation. At the same time, innovation cannot be confined to T2 COPD: most patients (an estimated 60–80%) fall outside this biology, and novel mechanisms, alongside earlier intervention in GOLD 1–2 disease, will be essential if the goal is to prevent progression rather than react to advanced disease. Even with these open questions, the direction of travel is unmistakable: COPD therapeutics is moving from empiricism toward endotype-guided precision care. Clinicians should consider biologic therapy for COPD patients meeting these criteria: blood eosinophils ≥ 300 cells/µL confirmed on two occasions; ≥2 moderate or ≥ 1 severe exacerbation in past 12 months despite optimized triple inhaled therapy for ≥ 3 months; documented medication adherence and completion of non-pharmacologic interventions; chronic productive cough consistent with chronic bronchitis phenotype; and absence of asthma history or asthma-COPD overlap features. Dupilumab represents the preferred first-line option given superior efficacy across all outcome domains, with particular consideration for patients with elevated FeNO and a phenotype of chronic bronchitis. Mepolizumab may be considered in patients with or without chronic bronchitis accepting efficacy expectations focused on exacerbation reduction alone.

## Conclusions

The advent of biologic therapies is changing the treatment paradigm for eosinophilic COPD, supporting a precision-medicine approach in a historically heterogeneous disease [77, 78]. Dupilumab approval for COPD marked a turning point. Conceptually, this evolution is similar to the shift from IPF to PPF, where treatment is guided by shared biology rather than by diagnostic labels alone. In the same way, T2 inflammation is a shared endotype across asthma and a subset of COPD, so treatment decisions can be guided by biomarkers and mechanism, not only by disease category. Within this perspective, eosinophilic COPD should also be approached in a broader multidimensional framework that acknowledges the relevance of coexisting conditions beyond the lower airways. In particular, coexisting conditions such as chronic rhinosinusitis may identify a more complex pan-airway phenotype, contribute to symptom burden and exacerbation risk, and support a multidisciplinary model of care consistent with the “united airways” concept. As evidence expands, from phase 3 programs to real-world effectiveness, the challenge remains optimizing patient selection and refining biomarker-guided algorithms to maximize clinical benefit and improve patients outcomes.

## Key References

- Bhatt SP, Rabe KF, Hanania NA, Vogelmeier CF, Cole J, Bafadhel M, et al. Dupilumab for COPD with Type 2 Inflammation Indicated by Eosinophil Counts. N Engl J Med. 2023;389(3):205–214. 10.1056/NEJMoa2303951.
  - ○ This was the first phase 3 trial to show that dupilumab significantly reduced exacerbations in COPD patients with evidence of type 2 inflammation. It provided the first strong proof that biologic therapy could be effective in a selected eosinophilic COPD population.
- Bhatt SP, Rabe KF, Hanania NA, Vogelmeier CF, Bafadhel M, Christenson SA, et al. Dupilumab for COPD with Blood Eosinophil Evidence of Type 2 Inflammation. N Engl J Med. 2024;390(24):2274–2283. 10.1056/NEJMoa2401304.
  - ○ This confirmatory phase 3 study showed that dupilumab significantly reduced exacerbations and improved lung function and patient-reported outcomes in COPD patients with evidence of type 2 inflammation. It represents a key trial supporting targeted biologic therapy in eosinophilic COPD.
- Bhatt SP, Rabe KF, Hanania NA, Vogelmeier CF, Bafadhel M, Christenson SA, et al. Dupilumab for chronic obstructive pulmonary disease with type 2 inflammation: a pooled analysis of two phase 3, randomised, double-blind, placebo-controlled trials. Lancet Respir Med. 2025;13(3):234–243. 10.1016/S2213-2600(24)00409-0.
  - ○ This pooled analysis of BOREAS and NOTUS provides the most comprehensive estimate of dupilumab efficacy in type 2-high COPD. By integrating results across both pivotal trials, it strengthens the robustness and generalizability of the treatment effect and helps define dupilumab’s role in clinical practice.
- Sciurba FC, Criner GJ, Christenson SA, Martinez FJ, Papi A, Roche N, et al. Mepolizumab to Prevent Exacerbations of COPD with an Eosinophilic Phenotype. N Engl J Med. 2025;392(17):1710–1720. 10.1056/NEJMoa2413181.
  - ○ This pivotal phase 3 study demonstrated a significant reduction in exacerbations with mepolizumab in COPD patients with an eosinophilic phenotype. The findings support IL-5 inhibition as a therapeutic option in a selected subgroup of COPD.
- Arndal E, Sørensen AL, Lapperre TS, Said N, Trampedach C, Aanæs K, et al. Chronic rhinosinusitis in COPD: A prevalent but unrecognized comorbidity impacting health related quality of life. Respir Med. 2020;171:106092. 10.1016/j.rmed.2020.106092.
  - ○ This study provides clinically relevant evidence that chronic rhinosinusitis is a common and frequently underrecognized comorbidity in COPD. Importantly, it demonstrates an association between CRS and impaired quality of life, supporting the view that upper-airway disease contributes meaningfully to overall disease burden and should be considered in the multidimensional assessment of COPD.

## Data Availability

No datasets were generated or analysed during the current study.
